# Transvenous endocardial pacing with SelectSecure^™^ 3830 lead in pediatric patients: case series of two infants and a literature review

**DOI:** 10.1186/s12872-024-03820-3

**Published:** 2024-03-05

**Authors:** Chuan Yang, Jing Qi, Mahmood Alam, Deling Zou

**Affiliations:** Department of Cardiology, Shengjing Hospital of China Medical University, 36 Sanhao Street, Shenyang, 110004 China

**Keywords:** Infants, Endocardial pacing, 3830 lead, Lumenless, Right ventricular septal pacing

## Abstract

**Background:**

The SelectSecure™ 3830 lead is an innovative, lumenless, and thin active fixed lead with a nonretractable screw-in tip and a diameter of 4.1 Fr, making it the thinnest pacing lead available. Its high anti-extrusion properties and durability have shown favorable outcomes in cardiac pacing, especially in pediatric patients. The superfine design and easy implantation of the lead have rendered it a preferred choice in children, particularly in cases of congenital heart disease.

**Case presentation:**

This case series presents two infant patients who underwent transvenous endocardial pacing using the SelectSecure™ 3830 lead, along with a comprehensive literature review on the topic. The study followed the patients for 5 years and 3 years, respectively, and observed stable pacing parameters, indicating a positive therapeutic outcome and safety.

This article discusses the optimal age and body shape for transvenous lead implantation in infants and highlights the advantages and disadvantages of endocardial and epicardial pacing approaches. Although endocardial pacing offers several benefits such as minimal trauma, short hospital stay, and longer battery life, it may not be suitable for intracardiac shunts, and venous occlusion remains a concern. On the other hand, epicardial pacing may be considered for children with challenging endocardial access but comes with higher risk of lead failure and coronary artery compression.

This study emphasizes the importance of careful follow-up in pediatric patients with pacing, as lead failure can occur in young patients owing to growth and development, leading to syncope and battery depletion. The article also underscores the significance of selecting the appropriate pacing location to minimize the impact of cardiac function, with right ventricular septal pacing emerging as a preferable option.

**Conclusions:**

The SelectSecure™ 3830 lead presents a promising solution for transvenous endocardial pacing in pediatric patients with high degree atrioventricular block and bradycardia, ensuring safe and effective pacing as they grow and develop.

## Background

Implantation of permanent pacemakers in infants has become increasingly necessary for decades in current clinical practice. Pacing electrodes for such patients must work reliably for a life-long period of pacing. Despite the higher service lifetime requirements and a low pacing lead replacement rate in young children, [1] lead-related complications are common in pediatric patients. In a large study, [2] a total of 1,007 leads were implanted in 497 pediatric patients, with a failure rate of 15%, and 28% of patients experienced multiple failures. Another ten-year single-centre retrospective study on infant pacing [3] showed that a total of 323 leads were implanted in 167 patients, and the complication rate was 9.3%. Lead failure (6.2%) and infection (2.7%) were the most common lead complications. The significant increase in pacing threshold and lead breakage were the primary causes of lead failure. Therefore, it is crucial to select appropriate leads for pediatric patients to reduce failure rates.

The SelectSecure™ 3830 lead (Medtronic™, United States) consists of two primary elements: the insulator and the electrode conductor. The insulator is constructed with a dual-layer design, incorporating silicone as the inner layer and polyurethane as the outer layer. Meanwhile, the electrode conductor is crafted from a platinum alloy coated with titanium nitride. So, it has a small diameter and a reliable long-term sensing and pacing ability, making it a preferred choice to reduce the frequency of lead replacement. The 3830 lead has already demonstrated safety and effectiveness for pacing in pediatric patients [4, 5]. The objective of this case series is to present the safety, operability, and effectiveness of the 3830 lead in infant patients under two-year-old in our medical centre.

## Case presentation

### Case 1

**Fig. 1:**
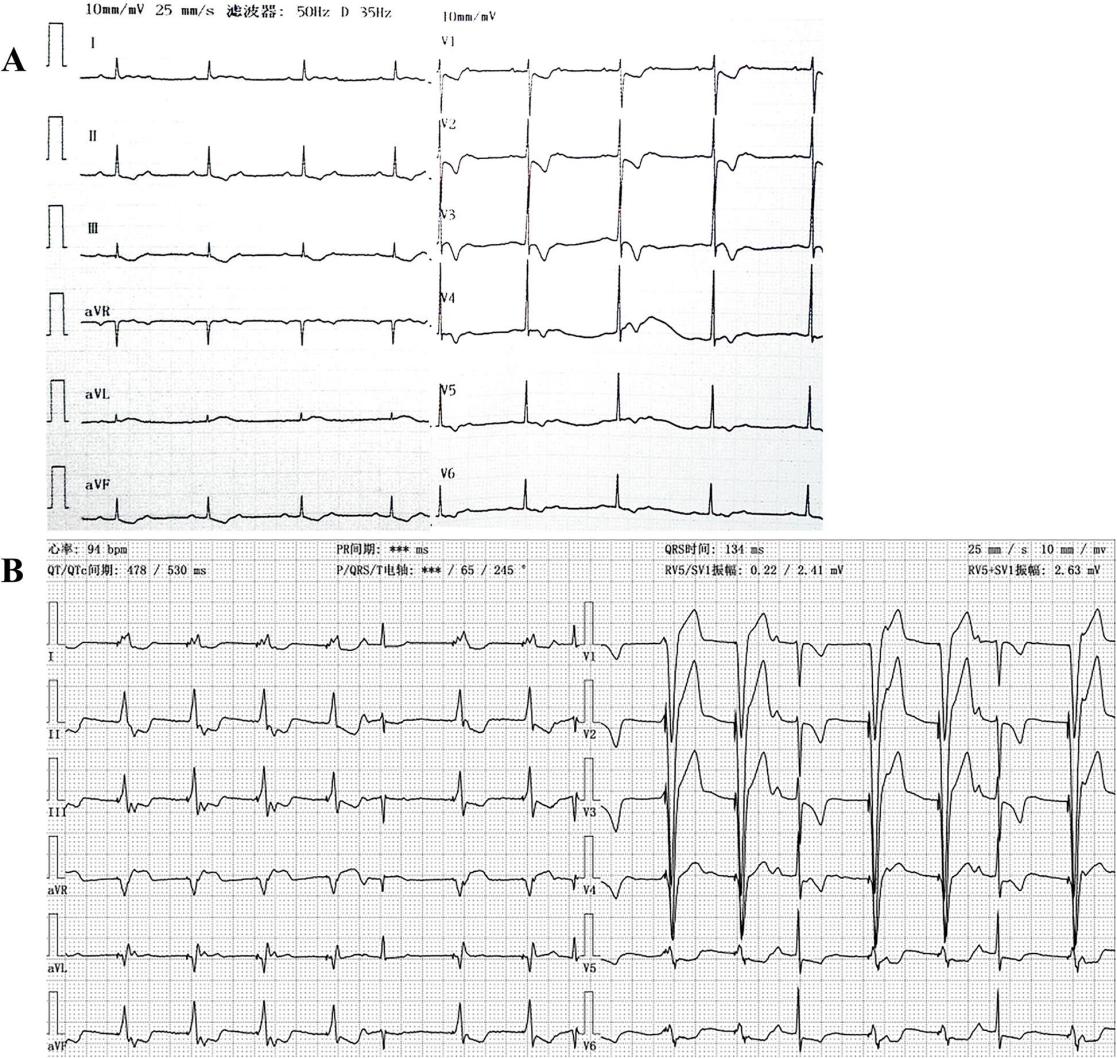
Upon admission ECG and pacing ECG. **A** Admission ECG: sinus rhythm, second-degree AVB (type II), ventricular rate was 67 bpm. **B** Postoperative pacing ECG: QRS duration was 134 ms

A 21-month-old female infant was admitted to the pediatric emergency room due to intermittent vomiting for one week and lethargy for one day. During the initial assessment, the patient was observed to have bradycardia, with a heart rate ranging from 60 to 70 bpm. Additionally, the electrocardiogram (ECG) revealed evidence of second-degree type II atrioventricular block (AVB), characterized by a 2:1 atrioventricular conduction ratio (Fig. 1A).

**Fig. 2:**
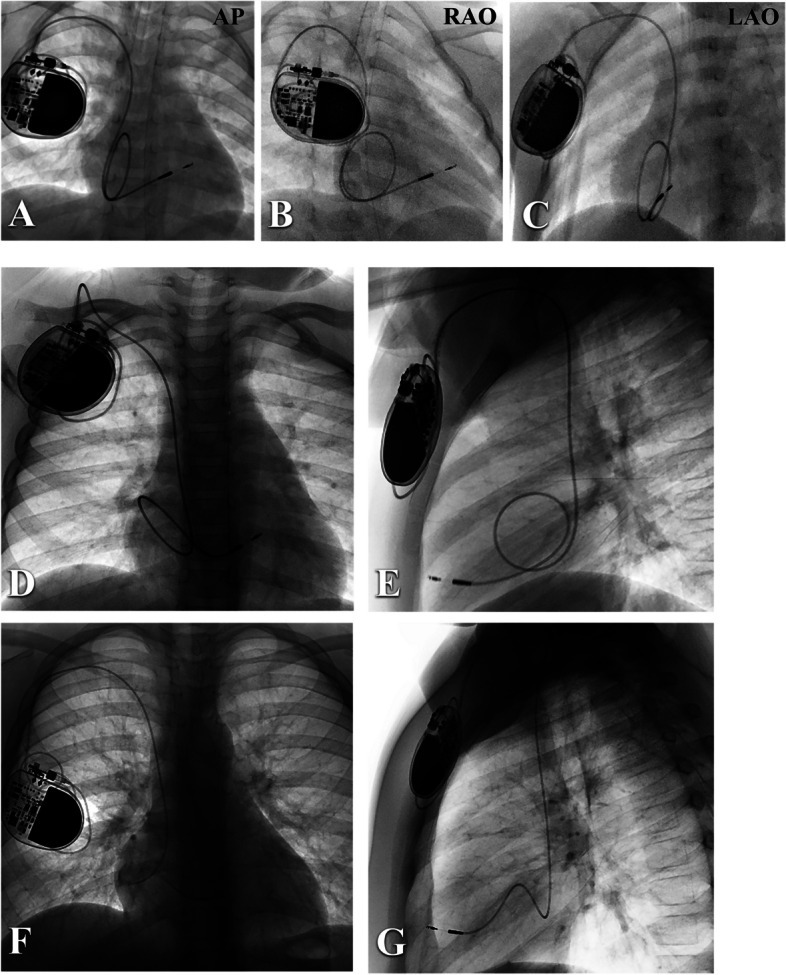
Radiology of the pacemaker implantation and follow-up. **A**,** B** and **C** are the X-ray images obtained during pacemaker implantation from AP, RAO, and LAO views. The 3830 lead was made into a redundant loop for a reservation of the growth in the RA. **D** and **E** are the anteroposterior and lateral chest radiographs at a 3-year follow-up. **F** and **G** are the anteroposterior and lateral chest radiographs in 5-year follow-up. The length of lead reserved in the RA is decreasing (AP: anteroposterior, RAO: right anterior oblique, LAO: left anterior oblique)

After admission, the patient experienced a sudden episode of ventricular tachycardia and cardiac arrest. Following cardiopulmonary resuscitation, the patient regained consciousness and normal heart function. The infant had a full-term natural birth, and her developmental milestones were consistent with those of other children her age. On the day of admission, her weight was 15 kg, and a family history revealed that the father had a permanent pacemaker implanted due to third-degree AVB. A routine examination was performed, and reversible causes were ruled out, but the AVB did not recover. One week later, the patient underwent permanent pacemaker implantation using a single-chamber pacemaker (Medtronic™ SESR01) and SelectSecure™ 3830 lead under general anesthesia. The puncture of the left subclavian vein was unsuccessful, so a catheter delivery sheath (Model C315-S5, Medtronic™) was inserted into the right ventricle (RV) via the right subclavian vein. The 3830 lead was threaded through the delivery sheath and navigated to the middle septum of the RV (Fig. 2A, B and C), where it was screwed in place. Intraoperative bedside echocardiography was implemented to determine the appropriate position of the electrodes. The sensing and capture thresholds were acceptable, with a sensed R wave of 12 mV and a threshold of 1.0 V / 0.4 ms. The pacing duration of the QRS complex was 134 ms (Fig. 1B). After removing the C315 sheath and cutting it into the right atrium (RA), the 3830 lead was looped in the RA to anticipate future growth needs.

One month after the operation, the pacemaker parameters were found to be optimal, with a threshold of 0.625 V / 0.4 ms, sensed R wave of 12 mV, and impedance of 475 Ω. A low limit frequency of 90 bpm was then set. A 3-year follow-up revealed that the position of the lead was normal (Fig. 2D and E). During the 5-year follow-up, the patient underwent an examination which showed that her growth and development were normal (Fig. 2F and G). Pacemaker programming confirmed stable lead parameters and normal pacemaker function. Although the chest radiograph indicated a reduced lead loop in the RA, the lead length was sufficient. Transthoracic echocardiography (TTE) showed normal cardiac function.

### Case 2

**Fig. 3:**
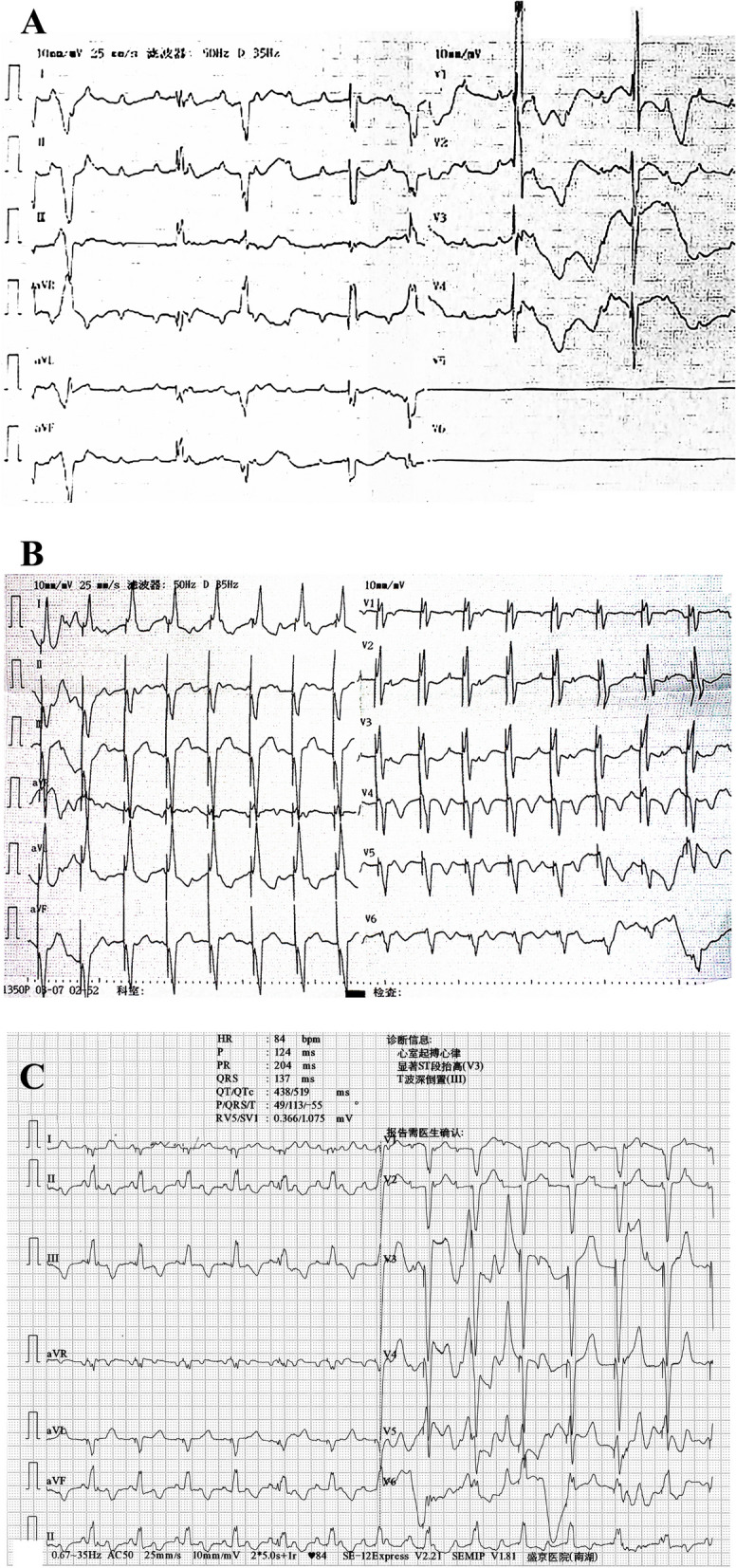
ECG at admission and ECG after pacing. **A**. ECG at admission showed third-degree AVB, and the ventricular rate was 42 bpm. **B**. ECG of epicardial pacing. **C**. ECG of endocardial pacing and the QRS duration was 137 ms

An 8-month-old male infant was admitted to the hospital following a transient loss of consciousness. Two days before admission, the baby experienced intermittent feeding and cyanosis around the mouth while crying. The following day, the baby had an episode of twitching, loss of consciousness, and paralysis of the limbs after crying, which lasted for 3–4 min. Concerned by these symptoms occurring multiple times, the parents sought medical treatment. During pregnancy, the fetal heart rate was found to be slow, ranging from 50 to 60 bpm. At birth, the baby weighed 2.5 kg and was delivered via a full-term cesarean section. However, after birth, the baby’s heart rate remained slow, leading to growth and developmental delays, and at 8 months of age, the infant was unable to sit alone. Upon admission, the baby weighed 5 kg, and ECG revealed a third-degree AVB with a ventricular rate of 42 bpm (Fig. 3A). TTE revealed a 6.4 mm left-to-right shunt in the atrial septum and a significant increase in the inner diameter of the RV by 19 mm, with an estimated pulmonary artery pressure of 50 mmHg. The left ventricular ejection fraction (LVEF) was 58%, and chest CT showed significant RV enlargement. A diagnosis of congenital third-degree AVB and atrial septal defect (ASD) was made.

Following admission, the infant received temporary pacing treatment, and subsequently, an epicardial lead (Medtronic™ 4965) was implanted under general anesthesia. The pulse generator (Medtronic™ SESR01) was placed beneath the rectus abdominis muscle. A VVIR pacing mode was set after implantation, with a low limit frequency of 100 bpm (Fig. 3B). Following the pacemaker implantation, the infant’s growth retardation significantly improved.

Nine months after the operation, the threshold of epicardial lead was 1.25 V / 0.4 ms, and the impedance was 243 Ω. So, there was no significant change in the parameters at the implantation (threshold of 1.1 V / 0.4 ms and impedance of 360 Ω).

However, 16 months after the operation, the child experienced sudden syncope and twitching at home. The emergency ECG revealed that ventricular pacing could not be captured, and the threshold of the epicardial lead had increased significantly. The ventricle could only be captured by increasing the output voltage to 6 V / 1.0 ms. After adjusting the parameters, the estimated service life of the pacemaker was approximately half a year. TTE showed a 2.7-mm ASD, a 19-mm inner diameter of the RV, and a 65% LVEF.

**Fig. 4:**
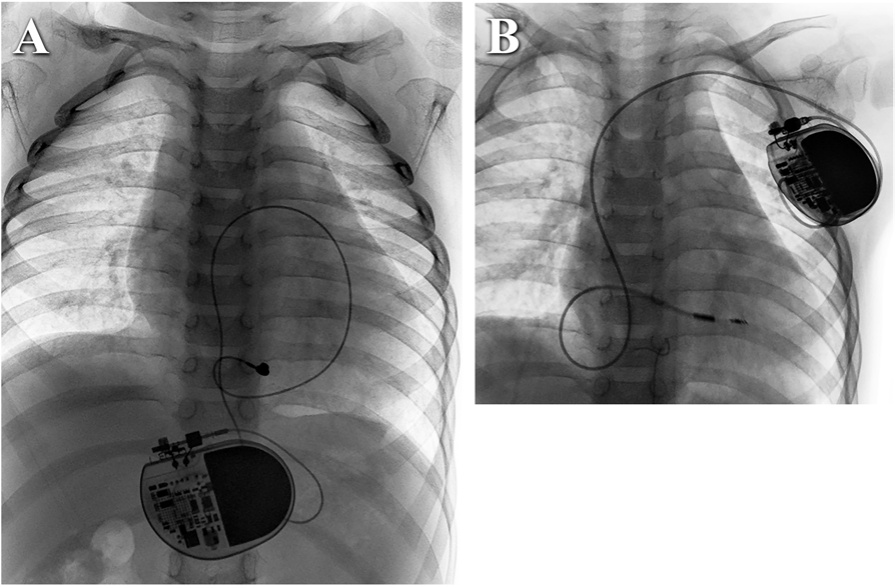
Chest X-ray imaging after epicardial and endocardial pacing. **A** The chest radiograph of an epicardial lead was implanted, and the pulse generator was buried beneath the rectus abdominis muscle. **B** The chest radiograph after endocardial pacing

To avoid further increased threshold of the epicardial lead and premature battery exhaustion, the pacemaker was replaced with a 3830 endocardial lead when the patient reached two years of age (16 months after epicardial pacing). At that time, the child weighed 11.5 kg. The 3830 lead was implanted through the left subclavian vein, and the pulse generator of the pacemaker (Medtronic™ SESR01) was placed under the pectoralis major muscle. The lead was coiled in a redundant loop in the RA to accommodate future growth needs. The epicardial lead and the previous pacemaker were removed. Echocardiography was utilized to confirm the placement of the lead anchored in RV and the redundant loop formed in the RA. The parameters were appropriately set (threshold 0.75 V / 0.4 ms, impedance 570 Ω), and the postoperative pacing durations of QRS complex was 137 ms. The pacemaker was programmed to VVIR pacing mode, with a lower limit frequency of 90 bpm (Fig. 3C). Figure 4 shows the chest X-ray images after epicardial and endocardial pacing, respectively.

During the 1.5 years of follow-up, the child’s growth and development were comparable to that of normal children, and the pacemaker functioned normally. The threshold and impedance of the lead remained stable.

**Fig. 5:**
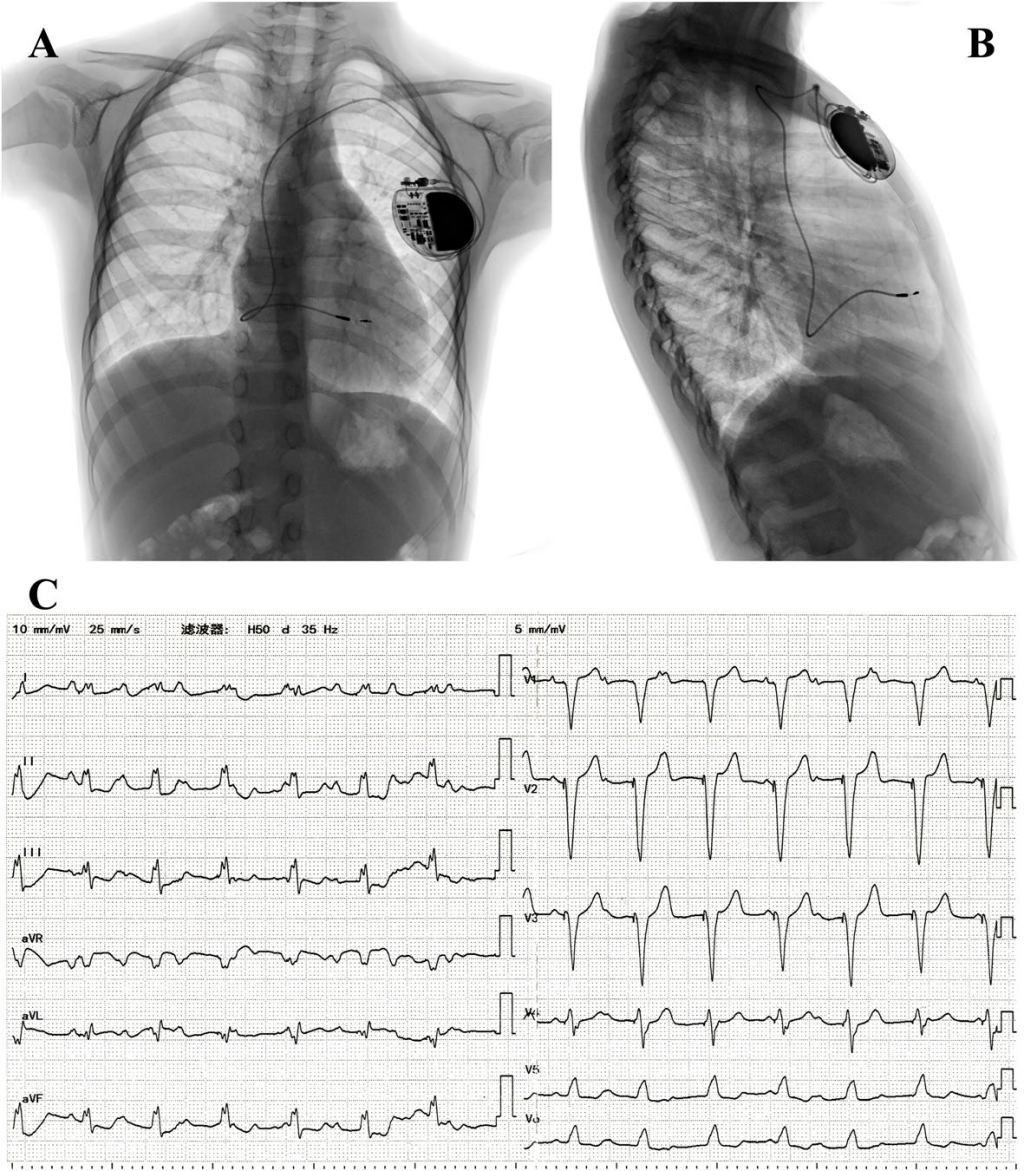
X-ray and ECG at the 3-year follow-up. **A**. Chest X-ray image from the AP view. **B**. Chest X-ray image from the lateral view. **C**. ECG displaying pacing rhythm with a QRS duration of 143 ms

At the most recent follow-up, conducted three years later, the patient reported no episodes of discomfort. A thorough examination, including a chest X-ray, indicated optimal positioning of the ventricular lead and generator. The ECG revealed a QRS complex pacing duration of 143 ms, as illustrated in Fig. 5. Notably, the ventricular pacing thresholds and impedance measurements were recorded at 0.88 V/ 0.4 ms and 468 Ω, respectively. TTE unveiled a 3.2-mm ASD, a 28-mm inner diameter of the RV, no tricuspid valve regurgitation, and a 54% LVEF.

## Discussion and conclusions

In this report, we presented two cases of infant patients who underwent transvenous endocardial implantation with SelectSecure™ 3830 pacing leads. Both patients were followed up for 5 years and 3 years, respectively, and their pacing parameters remained stable, indicating a successful treatment outcome with good efficacy and safety.

The 3830 lead is a lumenless active fixed lead with a thin diameter of 4.1 Fr, making it the thinnest pacing lead currently available. Despite its thinness, it exhibits high anti-extrusion properties, making it the preferred choice for transvenous pacing in children. Clinical research studies [6–8] have highlighted the advantages of the 3830 lead in cardiac pacing, including its durability, ease of implantation, and low complication rates, making it particularly suitable for pediatric patients and those with congenital heart disease in acute and subacute time periods [9]. A recent study extended this understanding, affirming the safety and feasibility of the 3830 transvenous pacing system in pediatric patients. The system demonstrated stable electrical performance during both acute and midterm periods, with the noteworthy absence of major complications [10]. 

The application of transvenous pacing in pediatric patients, especially those weighing less than 10 kg, [11] has demonstrated notable success in both short and long-term outcomes. However, the challenges arise from factors such as a diminutive body size, narrower venous diameter, and the ongoing process of somatic growth, which collectively constitute the most significant impediments to the widespread application of transvenous pacing in childhood [12]. Consensus on the established minimum body weight threshold for considering transvenous lead placement in pediatric patients is yet to be reached.

There is some debate about epicardial versus endocardial pacing in pediatric patients [13–16]. Endocardial lead implantation offers advantages such as non-thoracotomy, reduced trauma, shorter hospital stays, lower pacing thresholds, and longer battery life. However, this is not suitable for cases with intracardiac shunts. The disadvantage is that the incidence of venous occlusion is high, [17] and the physique is too small to be suitable. Additionally, multiple studies indicate a higher occurrence of complications such as thrombosis, pocket infection, tricuspid valve regurgitation, and infective endocarditis in pediatric cases [18–21]. As a general guideline, this method is recommended for children weighing over 10 kg, [22] and caution is advised in cases with anatomical abnormalities that impede lead placement through veins.

Compared to endocardial pacing, epicardial pacing is more invasive and has higher pacing thresholds, making it more susceptible to lead breakage and shorter battery life, and it can cause more coronary artery compression [23, 24]. It is suitable for a small number of children with difficult endocardial access. In 2013, a retrospective analysis [14] showed the results of a single centre over the past 26 years. A total number of 287 patients with congenital heart disease (CHD) with a median age of 5 (1–11 years) underwent cardiac pacing. Endocardial systems (Endo) were implanted in 117 patients, while epicardial systems (Epi) were implanted in 170 patients. The median follow-up period was 5 years (2–10 years). The pacing system failed in 29% of patients, which were 13% in Endo and 40% in Epi. Multivariate analysis showed a significantly higher risk of failure for Epi, a lower implant age, and a greater number of leads implanted. Endocardial systems of children with pacing showed significantly better results than Epi systems. Endocardial pacing also showed better long-term results than epicardial pacing. Another study [2] also suggested that the predictors of lead failure included young age at the time of implantation, CHD, and epicardial lead implantation. Epicardial lead is more likely to fail due to lead breakage or outlet block, while the transvenous endocardial lead mostly fails due to insulation layer fracture of lead displacement.

The second case presented in this report initially had epicardial pacing due to the child’s low body weight. However, after 16 months, a significant increase in the pacing threshold of the epicardial lead was observed, resulting in impaired pacing function and subsequent episodes of syncope. This case highlights the importance of choosing endocardial lead pacing whenever possible and the need for close follow-up to detect lead failure and avoid potential accidents due to pacemaker failure.

Implantation of the 3830 lead requires the assistance of a delivery sheath (Medtronic™ C315, C304 sheath), which facilitates precise placement of the pacing lead in the cardiac cavity. The C315 sheath, particularly the S4/S5 delivery sheath, is commonly used in infant patients. The 3830 lead’s thinness and anti-traction properties help reduce tricuspid regurgitation and subclavian vein compression syndrome. The 3830 leads have shown remarkable effectiveness and a minimal complication rate compared to conventional pacing leads [8, 25, 26]. 

To ensure successful and long-term pacing in infant patients, it is crucial to reduce lead failure and replacement rates while selecting appropriate pacing locations to minimize the impact on cardiac function. Although evidence suggests no significant difference in clinical results between right ventricular septum and apical pacing, right ventricular septal pacing offers advantages such as narrower QRS waves, better cardiac function maintenance, [2, 27] and a lower incidence of perforation, [28] making it more suitable for clinical practice. The delivery of the 3830 lead through the preformed sheath improves operability, [6] making it easier to select the precise ventricular septal pacing location and achieve satisfactory pacing effects. Both infants in our cases had the endocardial pacing lead successfully placed in the middle portion of the right ventricular septum, resulting in relatively short QRS complex durations, though not reaching the ideal durations of physiological pacing. However, further studies are essential to explore the feasibility, safety, and potential risks associated with bundle of His system pacing in infants.

In conclusion, the implantation process of the 3830-lead pacemaker is manageable and effective. For infant patients under two years old with high degree atrioventricular block and bradyarrhythmia, transvenous endocardial implantation of the 3830 lead into the right ventricular septum for pacing is a safe and viable option. The 3830 lead can be successfully and safely utilized in pediatric patients as they grow and develop.

## Data Availability

The datasets generated and/or analysed during the current case series are available in the GitHub repository, https://github.com/YangChuan80/CaseData_3830.

## Abbreviations

AVB: Atrioventricular block
ECG: Electrocardiogram
RV: Right ventricle
RA: Right atrium
TTE: Transthoracic echocardiography
LVEF: Left ventricular ejection fraction
ASD: Atrial septal defect
CHD: Congenital heart disease
